# Dalbavancin binds ACE2 to block its interaction with SARS-CoV-2 spike protein and is effective in inhibiting SARS-CoV-2 infection in animal models

**DOI:** 10.1038/s41422-020-00450-0

**Published:** 2020-12-01

**Authors:** Gan Wang, Meng-Li Yang, Zi-Lei Duan, Feng-Liang Liu, Lin Jin, Cheng-Bo Long, Min Zhang, Xiao-Peng Tang, Ling Xu, Ying-Chang Li, Peter Muiruri Kamau, Lian Yang, Hong-Qi Liu, Jing-Wen Xu, Jie-Kai Chen, Yong-Tang Zheng, Xiao-Zhong Peng, Ren Lai

**Affiliations:** 1 Key Laboratory of Animal Models and Human Disease Mechanisms, Chinese Academy of Sciences/Key Laboratory of Bioactive Peptides of Yunnan Province, KIZ-CUHK Joint Laboratory of Bioresources and Molecular Research in Common Diseases, National Resource Center for Non-Human Primates, Kunming Primate Research Center, and National Research Facility for Phenotypic & Genetic Analysis of Model Animals (Primate Facility), Kunming Institute of Zoology, Kunming, Yunnan 650107 China; 2 Institute of Medical Biology, Chinese Academy of Medical Sciences, Peking Union Medical College, Kunming, Yunnan 650031 China; 3 University of Chinese Academy of Sciences, Beijing, 100049 China; 4 Sino-African Joint Research Center, Chinese Academy of Science, Wuhan, Hubei 430074 China; 5 Kunming Institute of Botany, Chinese Academy of Sciences, Kunming, Yunnan 650201 China; 6 Guangzhou Institutes of Biomedicine and Health, Chinese Academy of Sciences, Guangzhou, Guangdong 510530 China; 7 Kunming National High-level Biosafety Research Center for Non-human Primates, Center for Biosafety Mega-Science, Kunming Institute of Zoology Chinese Academic of Sciences, Kunming, Yunnan 650107 China; 8 Institutes for Drug Discovery and Development, Chinese Academy of Sciences, Shanghai, 201203 China

**Keywords:** Molecular modelling, Mechanisms of disease

## Abstract

Infection with severe acute respiratory syndrome coronavirus 2 (SARS-CoV-2) has caused a pandemic worldwide. Currently, however, no effective drug or vaccine is available to treat or prevent the resulting coronavirus disease 2019 (COVID-19). Here, we report our discovery of a promising anti-COVID-19 drug candidate, the lipoglycopeptide antibiotic dalbavancin, based on virtual screening of the FDA-approved peptide drug library combined with in vitro and in vivo functional antiviral assays. Our results showed that dalbavancin directly binds to human angiotensin-converting enzyme 2 (ACE2) with high affinity, thereby blocking its interaction with the SARS-CoV-2 spike protein. Furthermore, dalbavancin effectively prevents SARS-CoV-2 replication in Vero E6 cells with an EC_50_ of ~12 nM. In both mouse and rhesus macaque models, viral replication and histopathological injuries caused by SARS-CoV-2 infection are significantly inhibited by dalbavancin administration. Given its high safety and long plasma half-life (8–10 days) shown in previous clinical trials, our data indicate that dalbavancin is a promising anti-COVID-19 drug candidate.

## Introduction

The infectious outbreak related to severe acute respiratory syndrome coronavirus 2 (SARS-CoV-2) was first reported in December 2019.^1,2^ With its extremely high dissemination potential, this virus has resulted in a global pandemic of coronavirus disease 2019 (COVID-19). By 11 November 2020, more than 50 million cases of SARS-CoV-2 infection have been reported, including 1,264,364 deaths in 214 countries. To date, however, no specific treatment or vaccine has been developed, highlighting the urgent need for antiviral drug and vaccine identification and development.^3^

Angiotensin converting enzyme 2 (ACE2), a dipeptidyl-carboxypeptidase type I integral membrane protein, is considered a therapeutic target for COVID-19 patients.^4^ Extensive studies have demonstrated that ACE2 is a critical receptor for coronavirus infections, including severe acute respiratory syndrome coronavirus (SARS-CoV) which emerged 17 years ago.^5^ SARS-CoV attaches to the host ACE2 receptor and then enters target cells by using the virus spike protein. At the genomic level, SARS-CoV-2 bears an 82% sequence resemblance to SARS-CoV.^6^ Their receptor binding domains (RBD) are conserved, suggesting that they may share a common host–cell ACE2 receptor. Several cryo-electron microscopy studies have demonstrated that the SARS-CoV-2 spike protein directly binds to ACE2 with high affinity.^7^ Recently, soluble human ACE2 has been found to block the early stages of SARS-CoV-2 infection in engineered human tissues, further indicating that targeting ACE2 may be an effective strategy for the development of antiviral medicines.^8^ However, no definite evidence from large-scale clinical studies has proved the efficacy of ACE2 inhibitors/angiotensin receptor blockers for treating COVID-19 patients.^9^ As a potential inhibitor of RNA-dependent RNA polymerase, Remdesivir is considered one of the most promising antiviral agents, despite relatively low efficacy observed in large-scale studies.^10–12^

To cope with the emerging need for antiviral drugs, drug repurposing can be an efficient strategy to treat novel diseases with minimal or no side effects. Here, based on virtual screening, we identified an approved lipoglycopeptide antibiotic, dalbavancin, with the potential to block spike protein–ACE2 interaction. In vitro studies indicated that dalbavancin showed a significant ability to inhibit SARS-CoV-2-induced cytopathic effects (CPEs) on Vero E6 cells. Furthermore, dalbavancin administration significantly reduced viral loads and pneumonia in both mouse and rhesus macaque models.

## Results

### Screening potential inhibitors of SARS-CoV-2-ACE2 interactions

**Fig. 1:**
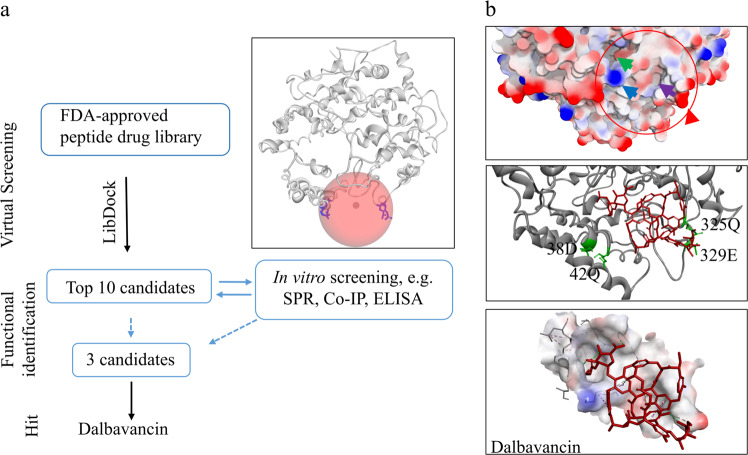
Screening potential inhibitors of SARS-CoV-2-ACE2 interactions. **a** Workflow indicating identification of ACE2-binding peptides. After virtual screening, for each high-scoring candidate peptide, in vitro experiments were performed to validate its binding ability to ACE2. Picture represents 3D structure of ACE2 (PDB ID: 3D0G) and binding site (red sphere) for virtual screening. Key residue side chains are shown in blue. **b** Top 3D structure represents surface analysis of the binding site of ACE2 with SARS-COV-2 spike protein. Red circle marks binding site of ACE2 with SARS-COV-2 spike protein. Red and blue arrows show high charge areas in pocket by creating an interpolated charge surface; green and purple arrows point to porose area and hydrophobic domain, respectively. Middle 3D complex structure represents interaction between protein ACE2 and peptide drug dalbavancin after molecular dynamics simulation. Red structure represents dalbavancin, and green residues represent the four residues (Glu329, Gln325, Gln42, and Asp38) important for the binding of SARS-CoV-2 spike to ACE2. Lime, pale green, mint, and violet dashed lines in bottom picture represent hydrogen bonds, van der Waals bonds, Pi-Donor hydrogen bonds, and Pi-Alkyl bonds, respectively.

We identified/screened Food and Drug Administration (FDA)-approved peptide drugs that may inhibit the interaction between the SARS-CoV-2 spike protein and human ACE2. Co-crystallization Protein Data Bank (PDB) data^13^ of the SARS-CoV-2 spike protein and ACE2 revealed that four amino acid residues (Glu329, Gln325, Gln42, and Asp38, Fig. 1a) in ACE2 are important for the binding of the SARS-CoV-2 spike protein to ACE2. Therefore, we determined the location of the four amino acid residues as binding sites for virtual screening (Fig. 1). Conventional docking procedures were used for virtual screening of the FDA-approved peptide drug library. Ten polypeptide drugs (Supplementary information, Table S1) showed the ability to bind to the pocket region of ACE2, suggesting that these peptide drugs have the potential to inhibit SARS-CoV-2 spike–ACE2 interaction.

### Dalbavancin inhibits SARS-CoV-2 spike protein–ACE2 interaction by directly binding to ACE2

**Fig. 2:**
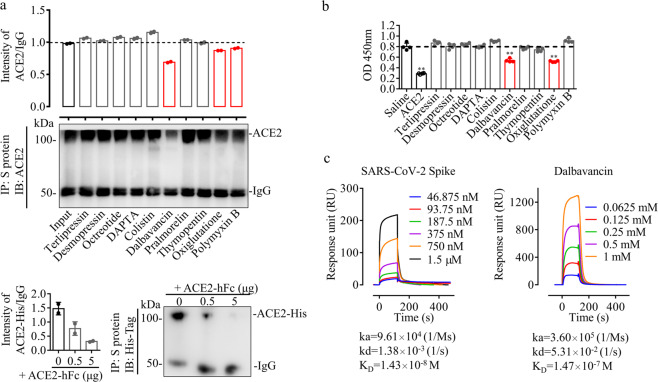
Dalbavancin inhibits binding of SARS-CoV-2 spike protein to ACE2 in vitro. **a** ACE2 (0.5 μg) and SARS-CoV-2 spike protein (0.5 μg) were mixed and treated with various candidate peptide drugs (10 μM). Co-precipitated proteins were identified by western blot analysis using anti-ACE2 antibody. Analyzed proteins are indicated on the right. For positive control (bottom), ACE2-His (0.5 μg) and SARS-CoV-2 spike protein (0.5 μg) were mixed and treated with various concentration of ACE2-hFc, and then co-precipitated proteins were identified by western blot analysis using anti-His-tag antibody. **b** ACE2 (2 μg/ml) was crosslinked to microplates by N-oxysuccinimide esters for ELISA. SARS-CoV-2 spike protein (10 ng/mL) and tested candidate drugs (1 μM) were incubated with ACE2, and extra un-crosslinked ACE2 protein (100 ng/mL) was used as a positive control. SARS-CoV-2 spike protein antibodies were used for chromogenic reaction. **c** Binding curves of immobilized human ACE2 with SARS-CoV-2 spike protein (left, positive control) and dalbavancin (right). Concentration-response SPR experiment showing binding of dalbavancin to ACE2 with an equilibrium dissociation constant (K_D_) of ~147 nM.

To verify the docking results, co-immunoprecipitation (co-IP) and enzyme-linked immunosorbent assays (ELISA) were conducted to confirm candidate peptide drugs that actively interrupt SARS-CoV-2 spike protein–ACE2 interaction in vitro. As illustrated in Fig. 2a, the SARS-CoV-2 spike protein and ACE2 exhibited successful co-precipitation. Furthermore, three candidate peptide drugs, including dalbavancin, oxiglutatione, and polymyxin B, suppressed the interaction between SARS-CoV-2 spike protein and ACE2, among which, dalbavancin showed the strongest inhibitory ability. Furthermore, ELISA was performed to determine the effects of the candidate peptide drugs on the interaction of the SARS-CoV-2 spike protein with ACE2 stabilized on microplates by N-oxysuccinimide esters. As illustrated in Fig. 2b, only two candidate peptide drugs (dalbavancin and oxiglutatione) inhibited the binding of the SARS-CoV-2 spike protein to ACE2. Surface Plasmon Resonance (SPR) was used for label-free detection of real-time monitoring of the binding interaction between dalbavancin and ACE2, which further confirmed that dalbavancin can directly bind to ACE2 (Fig. 2c). The binding response of dalbavancin to ACE2 was concentration dependent, with an equilibrium dissociation constant (K_D_) of ~147 nM.

### Dalbavancin potently inhibits SARS-CoV-2 in vitro

**Fig. 3:**
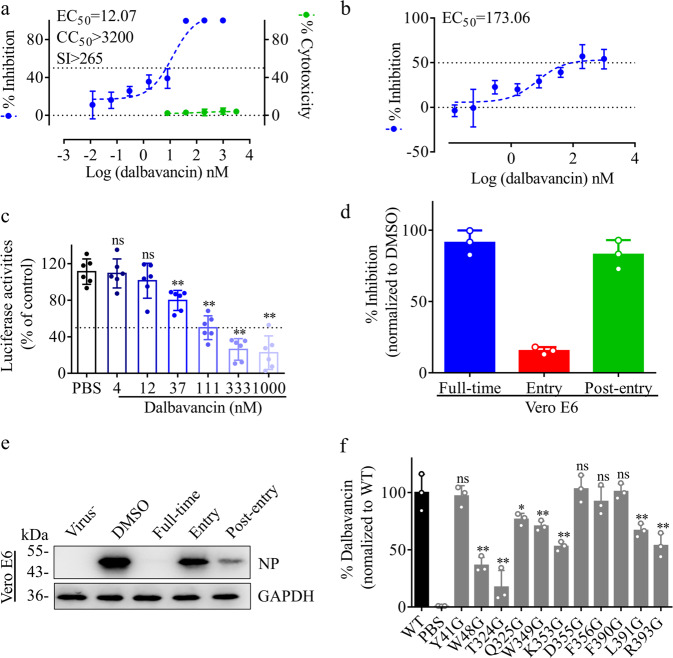
Antiviral activity of dalbavancin against SARS-CoV-2 in vitro. **a** Vero E6 cells were pre-incubated with indicated concentrations of dalbavancin for 1 h and cells were infected with SARS-CoV-2 at an MOI of 0.01. After 72 h, viral yield in cell supernatant was quantified by qRT-PCR. Cytotoxicity of these drugs to Vero E6 cells was measured by CCK-8 assays. Left and right y-axis of graphs represent mean % inhibition of virus yield and cytotoxicity of drugs, respectively. Experiments were performed in triplicate. CC_50_, half-cytotoxic concentration; SI, selectivity index. **b** After 1 h pre-incubation with different doses of dalbavancin, Caco-2 cells were infected with SARS-CoV-2 at an MOI of 0.01 for 72 h. The viral yield in the cell supernatant was quantified by qRT-PCR. **c** Inhibition of SARS-CoV-2 spike pseudo-virion entry by dalbavancin. HEK293/hACE2 cells were pre-incubated with indicated concentrations of dalbavancin, and then SARS-CoV-2 pseudo-virions were added to HEK293/hACE2 cells. At 48 hpi, transduction efficiency was measured according to luciferase activities. Means ± SD from at least three independent experiments with technical triplicates are shown. **d**, **e** Time-of-addition experiment of dalbavancin. For “Full-time” treatment, Vero E6 cells were pre-treated with testing drug (1 μM) for 0.5 h, and virus was then added to allow attachment for 1 h. Afterwards, the virus–drug mixture was removed, and the cells were cultured with dalbavancin (1 μM) at 24 hpi. For “Entry” treatment, dalbavancin (1 μM) was added to the cells for 0.5 h before viral attachment, and at 1 hpi., the virus–drug mixture was replaced with fresh culture medium and maintained for 24 h. For “Post-entry” experiment, dalbavancin (1 μM) was added at 0.5 hpi, and maintained for 24 h. Cells were infected with SARS-CoV-2 at an MOI of 0.05, and virus yield in the infected cell supernatants was quantified by qRT-PCR (**d**) and NP expression in infected cells was analyzed by western blot (**e**). **f** ACE2 mutant (2 μg) was incubated with dalbavancin (10 μg), anti-ACE2 antibody (1 μg), and protein A agarose (40 μL) overnight. After co-IP treatment, dalbavancin was analyzed by LC-MS/MS. Wild-type ACE2 (WT) and PBS were used as positive and negative control, respectively. Statistical significance was measured by two-way analysis of variance (ANOVA) compared with control group. ns, not significant, **P < 0.05, **P < 0.01.

Dalbavancin is shown to inhibit SARS-CoV-2 spike protein–ACE2 interactions by directly binding to ACE2. Its ability to reduce the CPEs of SARS-CoV-2 infection was assayed using the Vero E6 cell line. As shown in Supplementary information, Fig. S1a, at 72 h post-infection (hpi), most cells died due to severe CPEs caused by the virus. As a positive control, administration of 4 μM Remdesivir significantly inhibited the CPEs with little cell death. Dalbavancin was found to significantly reduce the CPEs caused by the viral infection (Fig. 3a). Efficacies were evaluated by quantification of viral copy numbers in the cell supernatant via quantitative real-time PCR (qRT-PCR). According to the dose–effect curve (Fig. 3a), the concentration for 50% of maximal effect (EC_50_) was ~12 nM. A recent study showed that TMPRSS2 is important for SARS-CoV-2 entry into cells.^14^ Dalbavancin shows a little ability to inhibit cathepsin L.^15^ Our current assay also indicated that 400 μM dalbavancin only inhibited ~40% enzymatic activity of cathepsin L, while the positive inhibitor cathepsin L inhibitor I (CTSL, MG-101, 20 μM) could inhibit ~100% the enzymatic activity (Supplementary information, Fig. S1b). We tested the anti-viral effect of dalbavancin using TMPRSS2-expressing cell line (Caco-2), finding that the EC_50_ on Caco-2 was higher than that in Vero E6 cells (Fig. 3b). We next determined whether dalbavancin can block SARS-CoV-2 pseudo-virions from entering the cell through binding to ACE2. Dalbavancin showed significant inhibition of SARS-CoV-2 pseudo-virion entry into HEK293/hACE2 cells, with an IC_50_ of ~53 nM (Fig. 3c). Time-of-addition assay showed that dalbavancin efficiently functioned at stage full-time and post-entry in Vero E6 cells. Weak inhibition (~20%) was observed at stage entry in Vero E6 cells (Fig. 3d, e). These results indicated that dalbavancin is a competitive inhibitor of ACE2, and the drug showed the most potent antiviral effect when it was continually maintained in medium.

To investigate the interacting sites of dalbavancin on ACE2, liquid chromatography-mass spectrometry (LC-MS/MS) linked co-IP was used to measure the binding affinity of ACE2 mutants with the drug. According to the docking model in Fig. 1b, eleven residues were predicted to be important for ACE2–dalbavancin interaction. Mutagenesis assays indicated seven of them (48W, 324T, 325Q, 349W, 353K, 391L, and 393R) are important for the interaction. (Fig. 3f; Supplementary information, Fig. S1c).

### Dalbavancin inhibits SARS-CoV-2 replication and prevents histopathological injuries caused by virus infection in a mouse model

**Fig. 4:**
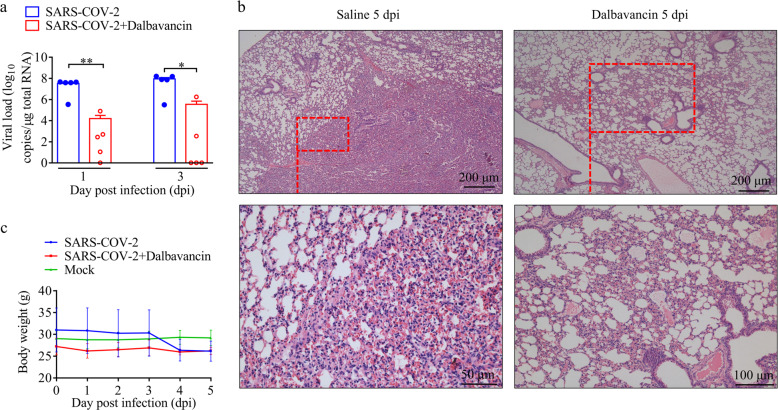
Dalbavancin inhibits SARS-CoV-2 replication and alleviates pneumonia in hACE2 mice. **a** hACE2 mice (*n* = 30) were infected with SARS-CoV-2 (100 μL by intranasally, 1 × 10^7^ TCID_50_) and their lungs were harvested for viral load analysis at 1, 3, and 5 dpi, respectively (at 5 dpi, both saline and dalbavancin groups had no virus detected). **b** Histopathology of lungs from saline-treated and dalbavancin-treated mouse groups (5 dpi). **c** Effects of dalbavancin on body weight of hACE2 mice after SARA-CoV-2 infection. Means ± SD are shown. Statistical significance was measured by two-tail *t-*test compared with control group. *P < 0.05, **P < 0.01.

As observed in other mouse models, mice infected with SARS-CoV-2 displayed transient infection and viral replication, but little virus was found at 72 hpi.^16^ At 24 hpi and 72 hpi, the viral load in the lungs of the control group was ~10^7^ and ~10^5^ copies/μg total RNA, respectively, whereas that in mice treated with a single dose of dalbavancin (130 mg/kg intraperitoneal administration at day 0) decreased to ~10^3^ and ~10^2^ copies, respectively (Fig. 4a). Thus, dalbavancin administration almost completely inhibited viral replication. Histopathological examination of the lungs indicated that most of mice in the control group showed typical interstitial pneumonia, characterized by infiltration of significant macrophages and lymphocytes into the alveolar interstitium, and accumulation of macrophages in alveolar cavities. In contrast, dalbavancin administration showed significant protective effects and prevented histopathological injuries caused by virus infection, with only mild histopathological injuries (Fig. 4b). Viral infection caused a decrease in mouse body weight, while dalbavancin administration rescued this decrease (Fig. 4c), with little effect on body temperature (Supplementary information, Fig. S2).

### Dalbavancin shows significant anti-SARS-CoV-2 effects in rhesus macaque model

**Fig. 5:**
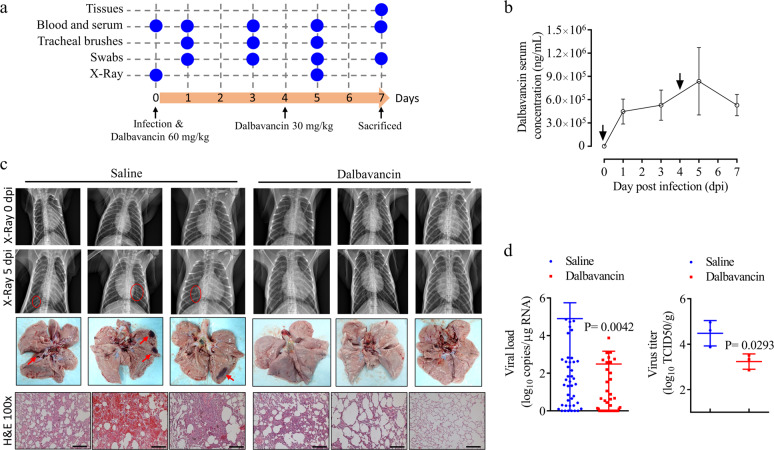
Dalbavancin shows significant anti-SARS-CoV-2 effects in rhesus macaque model. **a** Schematic of rhesus macaque model of SARS-CoV-2 infection. Macaques were randomly assigned to either the saline group (*n* = 3) or dalbavancin group (*n* = 3, administrated by intravenous infusion over 0.5 h) and inoculated with SARS-CoV-2 (1 × 10^7^ TCID_50_) via intratracheal (300 μL) and intranasal (200 μL) administration. At 7 dpi, animals from each group were euthanized and necropsied for virological and pathological assays. **b** Dalbavancin concentration analysis in serum of rhesus macaques infected with SARS-CoV-2. Dalbavancin was administered twice (two arrows, first 60 mg/kg, second 30 mg/kg) for 7 d. Dalbavancin showed a long half-life in serum. Means ± SD are shown. **c** Top, radiographs collected from each animal taken on 0 and 5 dpi. Areas of pulmonary infiltration are marked with a red circle. Middle, lungs from vehicle- or dalbavancin-treated monkeys. Dalbavancin-treated animals showed no visible pathological changes (red arrows). Bottom, histological analysis was performed on seven lung lobes from three animals per treatment group and representative images were chosen. Severe subpleural interstitial pneumonia and congestion were observed in three out of three vehicle-treated animals. Minimal (2/3) and moderate (1/3) subpleural interstitial pneumonia was observed in three dalbavancin-treated animals. Scale bars, 250 μm. **d** Left: Dalbavancin reduced viral loads in tissues collected from all seven lung lobes at 7 dpi from rhesus macaques infected with SARS-CoV-2. Right: Dalbavancin reduced virus titers in tissues collected from all seven lung lobes at 7 dpi from rhesus macaques. The seven lung lobe tissues (0.03 g) of one monkey were mixed and homogenized for virus titer test. Statistical significance was measured by two-tail *t*-test compared with control group.

Several non-human primate models, including cynomolgus and rhesus macaques, have been investigated as potential animal models for COVID-19. These SARS-CoV-2-infected non-human primates all displayed productive infection and clinical signs, ranging from asymptomatic in cynomolgus macaques to moderate disease in rhesus macaques.^17–22^ Of note, rhesus macaques have also been used to evaluate the neutralizing effects of adenovirus-vectored vaccines, DNA vaccine candidates, and Remdesivir administration against COVID-19.^21,23–25^ We next evaluated the anti-COVID-19 function of dalbavancin using rhesus macaques. Animals were randomly assigned to two groups (*n* = 3) for intratracheal and intranasal inoculation with SARS-CoV-2, respectively. Whole blood, serum, swabs, radiographs, and tracheal brushes were collected for several physiological and biochemical tests in rhesus macaque (Fig. 5a; Supplementary information, Fig. S3 and Tables S2, S3). The serum concentration of dalbavancin in the treated group was measured by LC-MS/MS, showing a long half-life of 5–7 days (Fig. 5b). Radiographic pulmonary infiltrates are one of the hallmarks of COVID-19 in humans. Here, radiographs taken on days 0 and 5 post-infection (dpi) showed significantly less severe pulmonary infiltration in macaques treated with dalbavancin compared to the saline control group (Fig. 5c, top). All animals were euthanized at 7 dpi and necropsies were performed. No significant pulmonary lesions were observed in the dalbavancin-treated group. In contrast, varying degrees of lung lesions at the gross pathological scale were observed in the control macaques (Fig. 5c, middle). The viral load and virus titer (Fig. 5d) were significantly lower in the lungs of dalbavancin-treated animals than that of the saline controls. Histological evaluation showed severe (3/3) subpleural interstitial pneumonia and minimal (3/3) infiltration of neutrophils and congestion (3/3) in the vehicle-treated animals. However, only minimal (2/3) and moderate (1/3) subpleural interstitial pneumonia and minimal (1/3) infiltration of neutrophils were observed in the three dalbavancin-treated animals (Fig. 5c, bottom). Meanwhile, dalbavancin reduced the amount of cytokines (IL-8 and MCP-1) in rhesus macaque lungs (Supplementary information, Fig. S4). These results further confirmed the anti-SARS-CoV-2 effects of dalbavancin.

## Discussion

In coping with public health emergencies, virtual screening of existing clinical drugs utilizing known crystal structures and binding sites can assist in quickly identifying potential therapeutic drugs. With the hope of finding candidate peptide drugs for SARS-CoV-2, we performed virtual screening of FDA-approved peptide drugs according to the structural characteristics of the binding pocket of the SARS-CoV-2 spike protein to ACE2.^13^ We showed that dalbavancin, a semisynthetic lipoglycopeptide antibiotic with a molecular weight of 1816.69 Dalton and a long plasma half-life of 5–7 days,^26^ which has been approved for the treatment of acute bacterial skin and skin structure infections,^27^ effectively prevented entry of SARS-CoV-2 into the cytoplasm, with an EC_50_ of ~12 nM. Thus, dalbavancin is a promising antiviral candidate for the treatment of SARS-CoV-2 infection. Furthermore, previous study has shown that dalbavancin inhibits SARS-CoV by acting as a protease inhibitor of cathepsin L,^15^ and its inhibitory effects on cathepsin L and its contribution to anti-SARS-CoV-2 were also confirmed in this study (Fig. 3b; Supplementary information, Fig. S1b).

At present, no drugs have shown significant efficacy in the clinical treatment of COVID-19 in large-scale studies. Therefore, there is an urgent need for drug repurposing to cope with the worsening trends of COVID-19 using approved drugs with minimal or no side effects. Avoiding the administration of non-steroidal anti-inflammatory drugs and targeting the ACE or angiotensin II type I receptor are advised for the treatment of COVID-19.^28^ Here, virtual screening indicated that dalbavancin potentially binds to ACE2 by interacting with the spike-binding region of the ACE2 protein. This was confirmed by SPR analysis, with a binding affinity of ~147 nM (Fig. 2c). Furthermore, co-IP, ELISA, and point mutation analysis also indicated that dalbavancin blocked the binding of the virus spike protein to ACE2 (Figs. 2a, b and 3f). Dalbavancin significantly blocked SARS-CoV-2 entry into the Vero cells and consequently reduced the CPEs caused by viral infection (Supplementary information, Fig. S1a).

The in vivo antiviral effects of dalbavancin were determined using both mouse and rhesus macaque models. Dalbavancin significantly inhibited viral replication directly, and thus promoted significant protective effects to prevent injuries caused by viral infection (Figs. 4 and 5).

The pharmacokinetic profile of dalbavancin indicates that it is highly protein-bound (>90%) in plasma, which contributes to its prolonged half-life and allows for once-weekly dosing.^27^ Here, we showed that dalbavancin also has a long half-life in rhesus macaque serum (Fig. 5b). Preclinical and clinical studies have shown that dalbavancin is well tolerated, without evidence of dose- or duration-related adverse effects.^29^ These merits strengthen the potency of dalbavancin as a promising antiviral candidate for the treatment of SARS-CoV-2 infection. Though animal models, which could faithfully recapitulate human SARS-CoV-2 pathogenesis, are still unavailable,^30,31^ the current anti-SARS-CoV-2 effects of dalbavancin observed in mouse and rhesus macaque models demonstrated sufficient potency and warrant further clinical trials.

## Materials and methods

### Virtual screening

The computational studies of ACE2 were based on the reference PDB model.^32^ For virtual screening, the ACE2 model was used for running a short (10 ns) molecular dynamics (MD) simulation to obtain the optimized model. A binding site sphere was generated (x: 42.43, y: −1.51, z: 122.69, r: 13.03, Fig. 1a) and used for screening the FDA-approved peptide drug library with LibDock. For MD simulations, the complexes were minimized, heated, and equilibrated using standard protocols. The production runs were carried out under the NPT ensemble with GROMACS v5.1.4 simulation software.^33^ The MD simulations were performed using an Amber99SB-ILDN force field for both protein and peptide using the GROMACS package. During MD simulations, all systems were solvated using the TIP3P^34^ water model in a periodic box, followed by the addition of 28 Na^+^ counter ions to neutralize the systems. The binding free energy of the complexes between peptide drug and ACE2 was calculated using CHARMm-based energies and implicit solvation methods. It is possible to estimate these free energies and thus calculate an estimate for overall binding free energy.^35^

### Cells and virus

The African green monkey kidney Vero E6 cell line (KCB 92017YJ) was obtained from the Conservation Genetics CAS Kunming Cell Bank (Kunming, China) and maintained in Dulbecco’s modified Eagle’s medium (DMEM, Gibco, USA) containing 10% fetal bovine serum (FBS) at 37 °C and in the presence of 5% CO_2_. A clinical SARS-CoV-2 isolate was propagated in the Vero E6 cells, and amplified SARS-CoV-2 was confirmed via qRT-PCR, sequencing, and transmission electron microscopy, and titrated via plaque assay (10^6^ pfu/mL). Infection experiments were performed in a biosafety level-3 (BLS-3) laboratory of the National Kunming High-level Biosafety Primate Research Center, Yunnan, China.

### Antiviral activities in vitro

To evaluate the antiviral efficacy of the testing samples, the Vero E6 or Caco-2 (TMPRSS2^+^) cells were pre-treated with different doses of drug for 1 h, and the virus (MOI = 0.01) was subsequently added to allow infection for 1 h. The virus–protein mixture was then removed, and cells were further cultured with fresh drug-containing medium. At 72 hpi, cell images were taken for analysis of CPEs and viral yield in the cell supernatant was then quantified by qRT-PCR. To evaluate the effects of dalbavancin on pseudo-virion entry, HEK293/hACE2 cells (Sino Biological, Beijing, China) were pre-treated with different concentrations of dalbavancin (HY-17586A, MedChemExpress, Monmouth Junction, NJ 08852, USA) for 1 h at 37 °C, then inoculated with SARS-CoV-2 S pseudo-virions (Sino Biological) in the presence of dalbavancin. The cells were lysed at 48 hpi and their luciferase activity was measured by Steady-Glo (Promega, Madison, WI, USA) according to the manufacturer’s instructions. Quantification of luciferase activity was performed using Infinite M200 (Tecan, Hombrechtikon, Switzerland).

### Cathepsin L activity assay

The inhibition of dalbavancin on cathepsin L enzymatic activity was measured using a Cathepsin L Activity Assay Kit (fluorometric, ab65306, Abcam) following the manufacturer’s protocol. Briefly, different concentrations of dalbavancin were added to the reaction buffer and mixed with cathepsin L (0.01 μL) provided in the kit. A positive control of cathepsin L inhibitor I (CTSL, MG-101, 20 μM) was provided in the kit. A total of 2 μL of 10 mM Ac-FR-AFC substrate was added to each well. The samples were incubated at 37 °C for 2 h. Fluorescence was measured using a multifunctional microplate reader (Cytation 3, BioTek, Winooski, VT, USA), at an excitation wavelength of 400 nm and an emission wavelength of 505 nm.

### ACE2 mutants and dalbavancin binding assay

Total 11 point mutated ACE2 plasmids (pCDNA3.1) were constructed and transfected in HEK293 cells. After expression, purification, and quantification, point mutated ACE2 proteins were used for dalbavancin binding assay. The binding of dalbavancin to ACE2 mutant proteins was measured by LC-MS/MS linked co-IP according to our previous method with a little modification.^36^ Briefly, dalbavancin (10 μg, HY-17586, MedChemExpress), point mutated ACE2 (2 μg), anti-ACE2 antibody (1 μg, 10108-T60, Sino Biological), and protein A agarose (40 μl, 15918014, ThermoFisher) were incubated overnight at 4 °C. After washing with PBS three times (5 min), dalbavancin was eluted with 70% ethanol and analyzed by LC-MS/MS.

### Anti-SARS-CoV-2 activities in humanized ACE2 (hACE2) mice

Murine studies were performed in an animal biosafety level 3 (ABSL3) facility using HEPA-filtered isolators. All procedures involving animals were reviewed and approved by the Institutional Committee for Animal Care and Biosafety in the Kunming Institute of Zoology (China) (SMKX-tz-20200415-03). All experiments complied with all relevant ethical regulations. For the animal experiments, male hACE2 mice were provided by the Guangzhou Institutes of Biomedicine and Health (China). After anesthetization by isoflurane, all hACE2 mice were inoculated intranasally with SARS-CoV-2 stock virus at a dose of 2 × 10^6^ TCID_50_. hACE2 mice intranasally inoculated with an equal volume of DMEM (Invitrogen, Carlsbad, USA) were used as the mock-infection control. For the drug-treated group, hACE2 mice were intraperitoneally injected with the dalbavancin (HY-17586, MedChemExpress, 130 mg/kg) 4 h before SARS-CoV-2 infection. The infected animals (control group *n* = 15, treatment group *n* = 15) were observed daily to record body weight and temperature. The mice were euthanized and dissected at 1, 3, and 5 dpi, respectively, to collect different tissues to screen virus replication and histopathological changes.

### Rhesus macaque model of SARS-CoV-2 infection

Primate studies were performed in an animal biosafety level 3 (ABSL3) facility using HEPA-filtered isolators. All procedures involving animals were reviewed and approved by the Institutional Committee for Animal Care and Biosafety in the Kunming Institute of Zoology (China) (IACUC20023). All experiments complied with all relevant ethical regulations. To evaluate the effects of dalbavancin treatment on SARS-CoV-2 disease outcome, we used a rhesus macaque model of SARS-CoV-2 infection. Six animals were randomly assigned to two groups and inoculated with a total of 500 μL (1 × 10^7^ TCID_50_) of SARS-CoV-2 by intratracheal (300 μL) and intranasal (200 μL) administration. The efficacy of therapeutic dalbavancin (HY-17586, MedChemExpress) treatment was tested in two groups of three rhesus macaques (adult males, 3–6 kg, and 4–7 year old). One group was treated with a loading dose of 60 mg/kg dalbavancin (by phleboclysis, 30 min), followed by a maintenance dose of 30 mg/kg at 4 dpi. The other group served as the infected controls and was administered with an equal dose volume of saline according to the same treatment schedule. All animal experiments and laboratory tests were performed after anaesthetization with Telazol. Nose, throat, and rectal swabs were collected twice daily during treatment. On 1, 3, and 5 dpi, tracheal brushes were collected for viral load tests.

Clinical parameters, such as body weight, temperature, pulse oximetry, blood pressure and respiration rate, were collected. Radiographs were taken at 0 and 5 dpi. The predetermined endpoint for this experiment was 7 dpi. Blood samples were collected from each animal for routine blood and serum biochemistry tests before euthanasia (i.v. 100 mg/kg pentobarbital sodium). Necropsies were then performed at 7 dpi to test viral loads and pathological lesions in the lungs and tracheas for viral RNA detection. Histopathological analysis of tissue slides was performed by a board-certified veterinary pathologist blind to group assignment of the animals. Flow cytometric analysis of cytokines in lungs, including IL-6, IL-10, CXCL10 (IP-10), IL-1β, IL-12p40, IL-17A, IFN-β, IL-23, TNF-α, IFN-γ, GM-CSF, CXCL8 (IL-8) and CCL2 (MCP-1) was performed by using LEGENDplex^TM^ Non-Human Primate (NHP) Inflammation Panel (Cat. No. 740389, Biolegend, San Diego, CA, USA) according to the manufacturer’s instruction. The virus titration of lung tissues was performed by end-point titration in Vero E6 cells. Tissues from 7 lung lobes of each monkeys were weighted, mixed, and homogenized in 1 mL DMEM. After the centrifugation for the supernatant, serial dilutions of supernatant were added onto Vero E6 cells to monitor the presence of CPE. Seven days after the inoculation, CPE was scored and the TCID_50_ was calculated and normalized per gram of lung tissue.

### qRT-PCR for detection of SARS-CoV-2 RNA

To evaluate viral loads, we used qRT-PCR as described previously.^37^ In brief, total RNA was extracted from swabs or tissue samples using a High Pure Viral RNA Kit (Roche, Germany) in accordance with the manufacturer’s instructions. TRIzol Reagent (Thermo, USA) was applied for RNA isolation using homogenized tissues. We followed the manufacturer’s protocols for one-step qRT-PCR using a THUNDERBIRD Probe One-Step qRT-PCR Kit (TOYOBO, Japan) to detect SARS-CoV-2 RNA. Previously reported primers targeting the N protein were used (5′-GGGGAACTTCTCCTGCTAGAAT-3′/5′-CAGACATTTTGCTCTCAAGCTG-3′ and probe FAM-TTGCTGCTGCTTGACAGATT-TAMRA-3′).^38^ In each run, serial dilutions of the SARS-CoV-2 RNA reference standard (National Institute of Metrology, China) were used in parallel to calculate copy numbers in each sample.

### ELISA

In brief, ACE2 (10 μg/mL) was diluted with phosphate-buffered saline (PBS, 2 mM KH_2_PO_4_, 8 mM Na_2_HPO_4_, 136 mM NaCl, 2.6 mM KCl, pH 7.4) and adsorbed onto a 96-well microplate (DNA-BIND, Corning; 100 μL per well) at 4 °C overnight. Plates were then blocked with 1% bovine serum albumin (BSA) in a wash solution (PBS buffer with 1% Tween-20 (v/v)) for 1 h at 37 °C. After washing the microplate with the buffer three times, spike (10 μg/mL) and tested drug (1 μM) or ACE2-hFc (10108-H02H, Sino Biological, China, for positive control) were added, followed by incubation for 1 h at 37 °C. The first anti-spike antibody (1:5000 dilution, 40150-R007, Sino Biological) was incubated in the solution for 1 h at 37 °C after washing the microplate with the buffer three times. After washing the microplate, a second anti-rabbit IgG antibody (1:10,000 dilution, horseradish peroxidase (HRP)-labeled, KPL, USA) was added, followed by incubation for 1 h at 37 °C. Color development was carried out with 100 μL of 3,3′,5,5′-tetramethylbenzidine (TMB, PR1200, Solarbio, China), and the reaction was stopped by the solution (1 M H_2_SO_4_). Absorbance at 450/630 nm was monitored on a plate reader.

### SPR analysis

BIAcore S200 (GE, USA) was used to analyze the interactions between the candidate peptide drugs and ACE2. Briefly, ACE2 (10108-H08B, Sino Biological Inc.) was first diluted (10 μg/mL) with 200 μL of sodium acetate buffer (10 mM, pH 5) and then flowed across the activated surface of a CM5 sensor chip (BR100012, GE, USA) at a flow rate of 5 μL/min, reaching a resonance unit (RU) of ~2000. The remaining activated sites on the chip were blocked with 75 μL of ethanolamine (1 M, pH 8.5). Serial concentrations of the tested drug (0.067, 0.125, 0.25, 0.5, and 1 mM) in PBS buffer were applied to analyze their interactions with immobilized ACE2 at a flow rate of 10 μL/min. The equilibrium dissociation constant (K_D_) for binding as well as the association (K_a_) and dissociation (K_d_) rate constants were determined using the BIA evaluation program (GE, USA).

### Co-IP and western blotting

Co-IP was performed as follows. ACE2 (1 μg) and the tested drug (10 μM) were incubated for 0.5 h in 0.5 mL of cold PBS buffer. ACE2-hFc was used for positive control. SARS-CoV-2 spike protein (1 μg) and its antibody (40150-R007, Sino Biological, China) were then added for other 0.5 h incubation, then mixed with 20 μL of protein A beads (10001D, ThermoFisher, USA) at 4 °C for 0.5 h with slow rotation to reduce non-specific binding. The immunoprecipitates were washed for five times with cold PBS buffer with 1% Tween-20 and re-suspended in 20 μL of sample buffer before being subjected to western blotting with antibodies against ACE2 (10108-T24, Sino Biological, China). Briefly, parts of the immunoprecipitated sample were resolved on an 8% SDS-PAGE gel, blotted, and probed for ACE2 using anti-ACE2 antibody or anti-His-tag antibody (12698S, CST, for positive control). The blots were incubated with anti-rabbit IgG HRP (KPL, USA) and imaged using electrochemiluminescence (ECL, ThermoFisher, USA).

## Supplementary information

Supplementary information, Fig. S1

Supplementary information, Fig. S2

Supplementary information, Fig. S3

Supplementary information, Fig. S4

Supplementary information, Tables

## Data Availability

The source data can be found at Mendeley Data (10.17632/8zw2r3npp7.1).
