# Supplementary material for: Dalbavancin binds ACE2 to block its interaction with SARS-CoV-2 spike protein and is effective in inhibiting SARS-CoV-2 infection in animal models
Source: Cell Res. 2020 Dec 1;31(1):17–24. doi: 10.1038/s41422-020-00450-0 (PMC7705431; doi:10.1038/s41422-020-00450-0)
Supplement: Supplementary file 1 — Supplementary information, Fig. S1 [file 41422_2020_450_MOESM1_ESM.pdf]

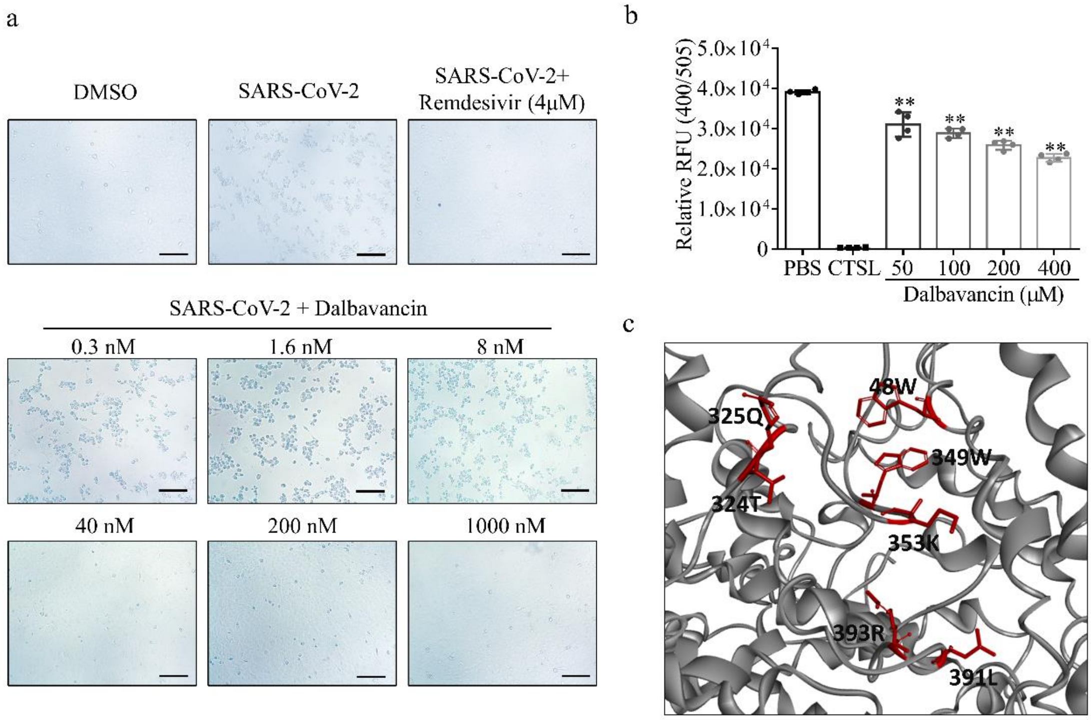

Supplementary information, Fig. S1: The antiviral activity of dalbavancin against SARS-CoV-2 in vitro. a Dalbavancin inhibits cytopathic effect (CPE) caused by SARS-CoV-2 infection in Vero-E6 cells. The bright field was used to evaluate CPE. Dalbavancin inhibited CPE and prevented Vero-E6 cell death after 72h post-infection with SARS-CoV-2. Scale bar, 25 μm. **b** Inhibition of Cathepsin L activity by dalbavancin. CTSL (20 μM, cathepsin L inhibitor I, MG-101) was used as a positive control. The mean ± SD from at least three independent experiments with technical triplicates is shown. Statistical significance was measured by ANOVA compared with control group. $^{**}P < 0.01$. **c** The 3D structure represents the binding site of dalbavancin on ACE2, point mutated residues (red) are affected the binding of dalbavancin to ACE2.
