# Supplementary material for: Dalbavancin binds ACE2 to block its interaction with SARS-CoV-2 spike protein and is effective in inhibiting SARS-CoV-2 infection in animal models
Source: Cell Res. 2020 Dec 1;31(1):17–24. doi: 10.1038/s41422-020-00450-0 (PMC7705431; doi:10.1038/s41422-020-00450-0)
Supplement: Supplementary file 2 — Supplementary information, Fig. S2 [file 41422_2020_450_MOESM2_ESM.pdf]

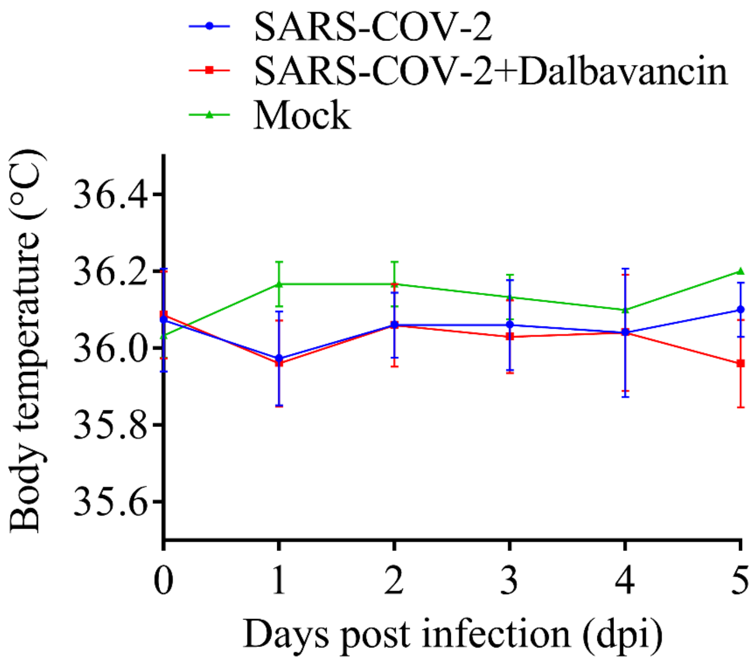

Supplementary information, Fig. S2: Effects of SARS-CoV-2 infection on body temperature in hACE2 mice. Body temperatures of mice were recorded for 5 d. hACE2 mice were intranasally administered with SARS-CoV-2, and ACE2-Mock mice (n=3) were used as the control. Neither SARS-CoV-2 nor dalbavancin had a significant effect on body temperature of mice.
