# Supplementary material for: Dalbavancin binds ACE2 to block its interaction with SARS-CoV-2 spike protein and is effective in inhibiting SARS-CoV-2 infection in animal models
Source: Cell Res. 2020 Dec 1;31(1):17–24. doi: 10.1038/s41422-020-00450-0 (PMC7705431; doi:10.1038/s41422-020-00450-0)
Supplement: Supplementary file 3 — Supplementary information, Fig. S3 [file 41422_2020_450_MOESM3_ESM.pdf]

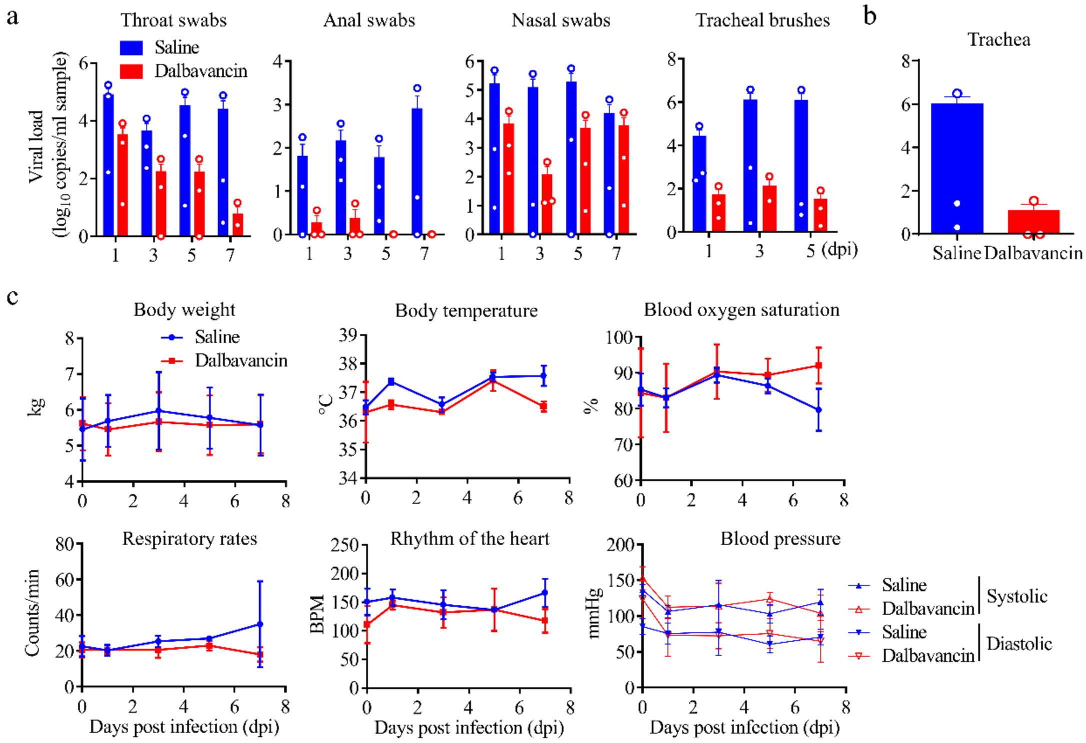

Supplementary information, Fig. S3: Effects of dalbavancin on viral loads and clinical signs in rhesus macaques infected with SARS-CoV-2. a Viral loads in swabs and tracheal brushes collected from rhesus macaques (n = 3). **b** Viral loads in tissues collected from tracheas on 7 dpi from rhesus macaques (n = 3). **c** Physical observations of rhesus macaques. Statistical analyses was performed using an unpaired t test.
