# Supplementary material for: Dalbavancin binds ACE2 to block its interaction with SARS-CoV-2 spike protein and is effective in inhibiting SARS-CoV-2 infection in animal models
Source: Cell Res. 2020 Dec 1;31(1):17–24. doi: 10.1038/s41422-020-00450-0 (PMC7705431; doi:10.1038/s41422-020-00450-0)
Supplement: Supplementary file 4 — Supplementary information, Fig. S4 [file 41422_2020_450_MOESM4_ESM.pdf]

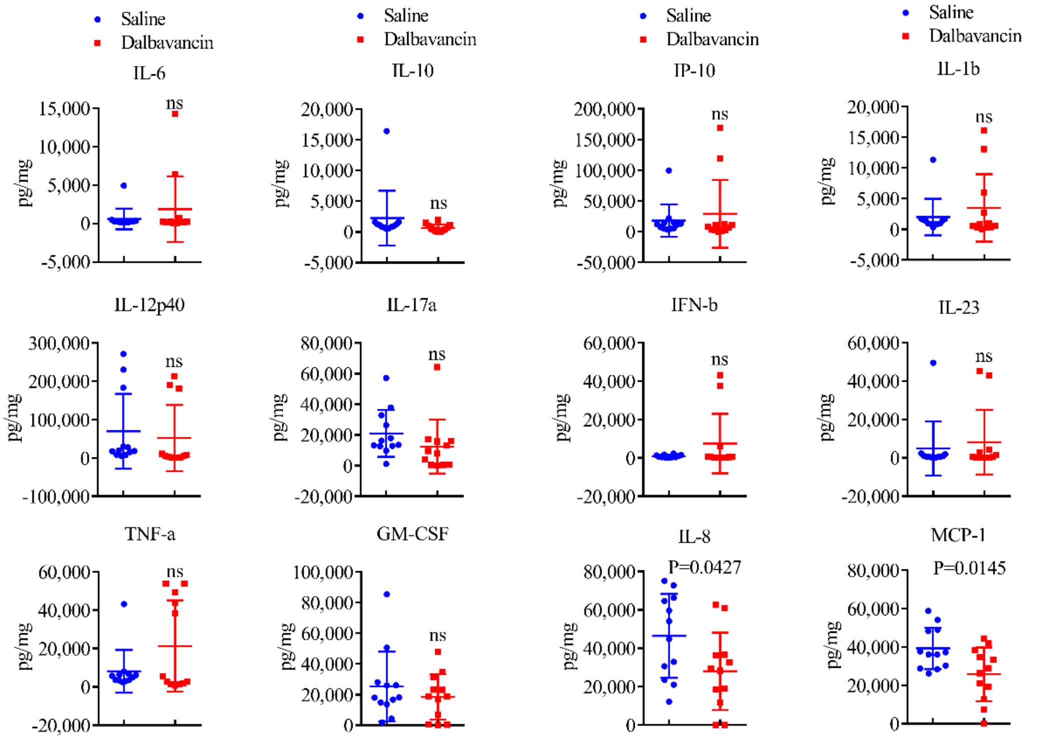

Supplementary information, Fig. S4: Effects of dalbavancin on cytokines changes in lung tissues of rhesus macaques infected with SARS-CoV-2 on 7 dpi. Dalbavancin reduced IL-8 and MCP-1 in tissues collected from all seven lung lobes at 7 dpi from rhesus macaques infected with SARS-CoV-2. Statistical analyses was performed using an unpaired t test.
