# Supplementary material for: Dalbavancin binds ACE2 to block its interaction with SARS-CoV-2 spike protein and is effective in inhibiting SARS-CoV-2 infection in animal models
Source: Cell Res. 2020 Dec 1;31(1):17–24. doi: 10.1038/s41422-020-00450-0 (PMC7705431; doi:10.1038/s41422-020-00450-0)
Supplement: Supplementary file 5 — Supplementary information, Tables [file 41422_2020_450_MOESM5_ESM.pdf]

Supplementary Tables

Table S1. Clinical peptide drugs with potential ability to inhibit ACE2-spike protein interactions

**Name**	**CAS**	**Reported target/function**	**Binding energy (kcal/mol)**
Terlipressin	14636-12-5	V1 receptors	-89.82
Desmopressin	16679-58-6	V1/2/3 receptors	-82.64
Octreotide acetate	79517-01-4	Hormone somatostatin	-82.61
DAPTA	106362-34-9	CCR5	-75.49
Colistin sulfate	1264-72-8	Anti-infection	-74.68
Dalbavancin	171500-79-1	Anti-infection	-68.78
Pralmorelin	158861-67-7	Growth hormone-releasing	-66.96
Thymopentin	69558-55-0	Immunology & HIV	-55.08
Oxiglutatione	27025-41-8	Detoxification	-55.07
Polymyxin B sulfate	1405-20-5	Anti-infection	-51.36

Table S2. Routine blood test results in rhesus macaques inoculated with SARS-CoV-2 with or without dalbavancin treatment.

Parameter		0 dpi	1 dpi	3 dpi	5 dpi	7 dpi
Saline	White blood cell count	8.900 ± 2.905	8.500 ± 1.305	6.333 ± 0.8192	7.967 ± 0.4333	8.200 ± 0.1000
	Lymphocyte count	1.900 ± 0.7638	2.967 ± 1.419	2.867 ± 0.7219	2.800 ± 0.7767	3.333 ± 0.4333
	Monocyte count	0.6333 ± 0.1453	0.7333 ± 0.1202	0.5667 ± 0.1453	0.7667 ± 0.1764	0.6000 ± 0.05774
	Neutrophil count	6.367 ± 3.299	4.800 ± 1.747	2.900 ± 0.4000	4.400 ± 0.7371	4.267 ± 0.5044
	Lymphocyte ratio	28.30 ± 13.78	35.10 ± 14.96	44.37 ± 6.471	34.53 ± 8.028	40.47 ± 5.725
	Monocyte ratio	7.733 ± 0.9025	8.567 ± 0.4702	8.800 ± 1.069	9.367 ± 1.761	7.600 ± 0.6807
	Neutrophil ratio	63.97 ± 14.68	56.33 ± 15.43	46.83 ± 6.919	56.10 ± 9.782	51.93 ± 5.948
	Red blood cell count	7.080 ± 0.04726	6.873 ± 0.3221	5.777 ± 0.2270	5.657 ± 0.3832	5.950 ± 0.1358
	Hemoglobin	170.3 ± 2.667	163.0 ± 2.517	142.7 ± 1.764	137.0 ± 7.550	141.0 ± 3.606
	Hematocrit	53.97 ± 1.757	51.93 ± 0.4333	42.67 ± 0.4096	41.60 ± 2.163	43.40 ± 1.124
	Mean corpuscular volume	76.37 ± 2.990	75.90 ± 2.804	74.13 ± 2.339	73.93 ± 2.697	73.10 ± 2.200
	Mean corpuscular hemoglobin	24.03 ± 0.5175	23.77 ± 0.8413	24.70 ± 0.6658	24.20 ± 0.7211	23.67 ± 0.7333
	Mean corpuscular hemoglobin concentration	315.3 ± 5.608	313.3 ± 4.055	334.0 ± 1.732	329.0 ± 2.517	324.3 ± 0.3333
	Red blood cell distribution width	13.53 ± 0.3283	13.97 ± 0.4910	12.10 ± 0.4041	12.07 ± 0.6173	12.17 ± 0.6119
	Platelet	550.7 ± 16.68	462.0 ± 31.58	353.0 ± 54.78	392.0 ± 75.74	446.7 ± 66.96
	Mean platelet volume	8.533 ± 0.3756	8.233 ± 0.3383	8.233 ± 0.3528	8.167 ± 0.3180	8.200 ± 0.3512

	Platelet distribution width	16.40 ± 0.2082	16.27 ± 0.1202	16.57 ± 0.1202	16.30 ± 0.1528	16.47 ± 0.2186
	Thrombocytocrit	0.4707 ± 0.03451	0.3820 ± 0.04204	0.2940 ± 0.05903	0.3237 ± 0.07435	0.3670 ± 0.06285
Dalbavancin	White blood cell count	9.300 ± 0.4933	8.433 ± 1.203	7.167 ± 0.4842	9.167 ± 2.338	8.233 ± 1.033
	Lymphocyte count	2.667 ± 0.7881	3.067 ± 0.7219	3.467 ± 0.3180	3.333 ± 0.5364	3.500 ± 0.2517
	Monocyte count	0.8000 ± 0.1528	0.9333 ± 0.2848	0.7333 ± 0.03333	0.7333 ± 0.2333	0.9000 ± 0.2646
	Neutrophil count	5.833 ± 0.9770	4.433 ± 1.486	2.967 ± 0.7424	5.100 ± 1.960	3.833 ± 0.5364
	Lymphocyte ratio	28.90 ± 8.883	38.93 ± 11.37	49.57 ± 8.137	39.53 ± 8.589	43.03 ± 2.298
	Monocyte ratio	8.500 ± 1.234	10.83 ± 1.991	10.20 ± 0.7638	7.567 ± 0.7126	10.83 ± 2.025
	Neutrophil ratio	62.60 ± 9.655	50.23 ± 9.672	40.23 ± 8.500	52.90 ± 7.881	46.13 ± 0.7446
	Red blood cell count	6.847 ± 0.2375	7.180 ± 0.1387	6.047 ± 0.1625	5.953 ± 0.1239	6.007 ± 0.3055
	Hemoglobin	166.3 ± 10.04	170.3 ± 9.955	149.0 ± 7.024	142.7 ± 4.410	141.3 ± 11.35
	Hematocrit	50.77 ± 3.230	53.23 ± 2.910	44.10 ± 1.637	43.17 ± 1.317	43.93 ± 3.483
	Mean corpuscular volume	74.20 ± 3.620	74.20 ± 3.747	73.17 ± 3.430	72.73 ± 3.524	73.13 ± 3.710
	Mean corpuscular hemoglobin	24.23 ± 1.135	23.67 ± 1.260	24.63 ± 1.302	23.97 ± 1.132	23.47 ± 1.132
	Mean corpuscular hemoglobin concentration	327.3 ± 1.202	319.3 ± 1.333	337.0 ± 3.786	330.3 ± 3.667	321.3 ± 1.764
	Red blood cell distribution width	13.73 ± 0.8667	13.80 ± 0.8185	13.93 ± 0.8007	13.43 ± 0.7753	13.97 ± 0.8950
	Platelet	650.0 ± 42.00	534.0 ± 49.90	480.7 ± 10.59	452.0 ± 24.58	545.3 ± 41.91
	Mean platelet volume	7.567 ± 0.2906	7.467 ± 0.2848	7.333 ± 0.08819	7.267 ± 0.2028	7.633 ± 0.5207
	Platelet	16.30 ±	16.37 ±	16.27 ±	16.23 ±	16.27 ±

distribution width	0.1528	0.06667	0.1333	0.1333	0.1333
Thrombocytocrit	0.4887 ± 0.01338	0.3957 ± 0.02281	0.3520 ± 0.004726	0.3273 ± 0.008969	0.4187 ± 0.05795

Data are mean ± SD.

Table S3. Blood biochemistry results in rhesus macaques inoculated with SARS-CoV-2 with or without dalbavancin treatment.

Parameter		0 dpi	1 dpi	3 dpi	5 dpi	7 dpi
Saline	Blood sugar mg/dL	144.7 ± 40.88	89.00 ± 13.43	50.33 ± 5.239	52.67 ± 6.766	56.33 ± 13.54
	Creatinine (CREA) mg/dL	0.7667 ± 0.1764	0.7000 ± 0.1528	0.5000 ± 0.05774	0.5333 ± 0.03333	0.5333 ± 0.03333
	Blood urea nitrogen (BUN) mg/dL	40.00 ± 8.185	34.33 ± 4.842	25.00 ± 1.155	19.67 ± 0.6667	18.00 ± 1.155
	BUN/CREA	52.67 ± 6.064	51.33 ± 4.096	49.00 ± 3.606	37.00 ± 3.055	34.00 ± 4.000
	Total protein g/dL	8.300 ± 0.1000	8.033 ± 0.1333	7.300 ± 0.1528	7.233 ± 0.06667	6.967 ± 0.2603
	Albumin (ALB) g/dL	4.033 ± 0.06667	3.733 ± 0.1202	3.267 ± 0.1202	3.300 ± 0.05774	3.167 ± 0.08819
	Globulin (GLOB) g/dL	4.233 ± 0.03333	4.367 ± 0.1856	4.033 ± 0.1453	3.967 ± 0.08819	3.833 ± 0.2028
	ALB/GLOB	0.9333 ± 0.03333	0.8667 ± 0.06667	0.8000 ± 0.05774	0.8333 ± 0.03333	0.8667 ± 0.03333
	Alanine aminotransferase U/L	29.67 ± 12.20	31.67 ± 7.126	53.67 ± 18.49	99.67 ± 42.48	59.00 ± 24.11
	Aspartate aminotransferase U/L	22.33 ± 11.61	45.33 ± 7.219	51.00 ± 7.550	52.33 ± 11.41	45.00 ± 10.58
	Cholesterol mg/dL	155.7 ± 15.86	109.0 ± 18.88	70.67 ± 2.728	84.00 ± 3.786	83.67 ± 1.667
	Triglyceride mg/dL	57.33 ± 9.838	54.00 ± 6.506	31.33 ± 19.13	48.67 ± 0.8819	52.00 ± 5.132
	Creatine kinase U/L	83.67 ± 22.30	349.7 ± 122.5	69.67 ± 6.489	92.67 ± 13.48	96.00 ± 28.01
	Lactate dehydrogenase U/L	792.7 ± 316.5	864.7 ± 154.7	1634 ± 532.9	1279 ± 541.7	1286 ± 301.0
	Lactate mmol/L	8.060 ±	8.907 ±	5.497 ±	4.683 ±	6.517 ±

		0.9829	1.583	1.066	0.8274	1.353
Dalbavancin	Blood sugar mg/dL	117.7 ± 23.31	72.33 ± 8.007	52.67 ± 4.842	53.00 ± 4.583	42.67 ± 4.667
	Creatinine (CREA) mg/dL	0.6000 ± 0.05774	0.6333 ± 0.1453	0.5333 ± 0.06667	0.5667 ± 0.03333	0.7000 ± 0.1528
	Blood urea nitrogen (BUN) mg/dL	50.67 ± 15.38	39.00 ± 8.083	27.67 ± 4.667	25.67 ± 4.702	24.33 ± 4.910
	BUN/CREA	94.67 ± 38.52	70.33 ± 26.27	55.33 ± 9.735	46.33 ± 5.548	41.33 ± 12.99
	Total protein g/dL	8.067 ± 0.1667	8.667 ± 0.1764	7.700 ± 0.2082	7.633 ± 0.3844	7.833 ± 0.3756
	Albumin (ALB) g/dL	3.633 ± 0.2028	4.033 ± 0.3528	3.467 ± 0.2333	3.400 ± 0.1528	3.933 ± 0.6888
	Globulin (GLOB) g/dL	4.400 ± 0.3786	4.600 ± 0.4933	4.233 ± 0.4333	4.200 ± 0.4359	3.900 ± 0.3786
	ALB/GLOB	0.8000 ± 0.1155	0.9000 ± 0.1732	0.8333 ± 0.1453	0.8000 ± 0.1000	1.067 ± 0.2728
	Alanine aminotransferase U/L	31.00 ± 6.658	39.67 ± 16.33	25.67 ± 9.563	44.67 ± 13.87	50.67 ± 26.34
	Aspartate aminotransferase U/L	16.00 ± 8.000	91.33 ± 58.34	40.33 ± 8.413	50.67 ± 11.68	55.50 ± 21.50
	Cholesterol mg/dL	171.0 ± 16.20	153.0 ± 15.95	89.33 ± 9.333	88.67 ± 8.212	107.0 ± 16.07
	Triglyceride mg/dL	38.67 ± 8.293	82.00 ± 42.00	60.33 ± 14.81	48.67 ± 0.8819	75.33 ± 38.40
	Creatine kinase U/L	189.3 ± 63.94	573.0 ± 370.5	141.0 ± 47.04	275.7 ± 47.68	552.7 ± 337.8
	Lactate dehydrogenase U/L	1544 ± 271.6	1771 ± 122.0	2249 ± 372.7	1650 ± 195.2	1932 ± 63.50
	Lactate mmol/L	6.787 ± 2.322	7.463 ± 2.455	6.500 ± 2.757	3.193 ± 0.5154	6.110 ± 2.954

Data are mean ± SD.
